# Long-Term Exposure to Fine Particulate Matter (PM2.5) Components and Precocious Puberty Among School-Aged Children: Cross-Sectional Study

**DOI:** 10.2196/62861

**Published:** 2025-02-07

**Authors:** Xuelian Zhou, Xiaochi Zhang, Guannan Bai, Guanping Dong, Xinyi Li, Ruimin Chen, Shaoke Chen, Rongxiu Zheng, Chunlin Wang, Haiyan Wei, Bingyan Cao, Yan Liang, Hui Yao, Zhe Su, Mireguli Maimaiti, Feihong Luo, Pin Li, Min Zhu, Hongwei Du, Yu Yang, Lanwei Cui, Jinling Wang, Jinna Yuan, Zhuang Liu, Wei Wu, Qi Zhao, Junfen Fu

**Affiliations:** 1 Department of Endocrinology, Children’s Hospital, Zhejiang University School of Medicine, National Clinical Research Center for Child Health, National Children’s Regional Medical Center, 3333 Binsheng Road, Hangzhou, 310051, China, 86 0571-86670013; 2 Department of Epidemiology, School of Public Health, Cheeloo College of Medicine, Shandong University, Jinan, China; 3 Shandong University Climate Change and Health Center, Shandong University, Jinan, China; 4 Department of Child Health Care, Children's Hospital, Zhejiang University School of Medicine, National Clinical Research Center for Child Health, Hangzhou, China; 5 Department of Endocrinology, Children’s Hospital of Fuzhou, Fujian Province, Fuzhou, China; 6 Department of Pediatrics, Maternal and Child Health Hospital of Guangxi Zhuang Autonomous Region, Nanning, China; 7 Department of Pediatrics, Tianjin Medical University Central Hospital, Tianjin, China; 8 Department of Pediatrics, The First Affiliated Hospital of Zhejiang University School of Medicine, Hangzhou, China; 9 Department of Endocrinology, Zhengzhou Children’s Hospital, Zhengzhou, China; 10 Department of Endocrinology, Beijing Children’s Hospital, Capital Medical University, National Medical Center for Children’s Health, Beijing, China; 11 Department of Pediatrics, Tongji Hospital, Tongji Medical College, Huazhong University of Science and Technology, Wuhan, China; 12 Department of Pediatrics, Wuhan Children’s Hospital, Tongji Medical College, Huazhong University of Science & Technology, Wuhan, China; 13 Department of Endocrinology, Shenzhen Children’s Hospital, Shenzhen, China; 14 Department of Pediatrics, The First Affiliated Hospital of Xinjiang Medical University, Urumqi, China; 15 Department of Pediatric Endocrinology and Inherited Metabolic Diseases, Children’s Hospital of Fudan University, Shanghai, China; 16 Department of Endocrinology, Children’s Hospital of Shanghai Jiaotong University, Shanghai, China; 17 Department of Endocrinology, Children’s Hospital of Chongqing Medical University, Chongqing, China; 18 Department of Pediatric Endocrinology, The First Bethune Hospital of Jilin University, Changchun, China; 19 Department of Endocrinology, Jiangxi Provincial Children’s Hospital, Nanchang, China; 20 Department of Pediatrics, The First Affiliated Hospital of Harbin Medical University, Harbin, China; 21 Department of Reproductive Medicine, Hospital of Jining Medical University, Jining, China; 22 Faculty of Health, Deakin University, Melbourne, Australia

**Keywords:** fine particulate matter, PM_2.5_, PM_2.5 _components, air pollution, precocious puberty, children, long-term exposure

## Abstract

**Background:**

The increasing incidence of precocious puberty is a major health challenge for Chinese children, while related risk factors remain less well explored. Exposure to ambient fine particulate matter (PM_2.5_) is a leading environmental hazard in China. Although certain components of PM_2.5_ have been reported to be endocrine disruptors for sex hormones, population-based evidence is still lacking on the association between PM_2.5_ exposure and precocious puberty in China.

**Objective:**

Based on a cross-sectional survey covering 30 cities in 2017 to 2019, this study was designed to explore the association between long-term exposure to PM_2.5_ and its 5 major components with precocious puberty in China and to check the potential modifying effects of family-related and personal factors.

**Methods:**

We included 34,105 children aged 6 to 9 years. We collected the 5-year average concentrations of PM_2.5_ and its 5 major components (sulfate, nitrate, ammonium, organic matter, and black carbon) in the area (at a spatial resolution of 0.1° × 0.1°) where each school was located. We used mixed effect logistic regression to estimate the effect sizes of the total mass of PM_2.5_ and each of its components on precocious puberty, and we examined the modifying effects of family-related and personal factors using an additional interactive term. A weighted quantile sum (WQS) regression model was applied to identify the weights of each component in explaining the effect size of the total mass of PM_2.5_.

**Results:**

We found that the odds ratio (OR) for precocious puberty per IQR increase in the concentration of total PM_2.5_ mass was 1.27 (95% CI 0.92-1.75) for the whole population, 2.12 (95% CI 1.27-3.55) for girls, and 0.90 (95% CI 0.62-1.30) for boys. Similarly, the effect sizes of the 5 major components were all substantial for girls but minimal for boys. Results of the WQS analysis showed that organic matter could explain the highest proportion of the effect of PM_2.5_, with the weight of its contribution being 0.71. Modification effects of family income and dietary habits were only observed in certain population subgroups.

**Conclusions:**

Long-term exposure to total PM_2.5_ mass was significantly associated with precocious puberty in girls, with organic matter identified as the major effect contributor. The results add evidence on the detrimental effects of PM_2.5_ on children’s development and growth.

## Introduction

Precocious puberty is an endocrine and metabolic disorder that may promote the development of pathophysiological or psychosocial outcomes in childhood and later life, including stunted growth, obesity, hypertension, type 2 diabetes, ischemic heart disease, stroke, and breast cancer [1]. Over the past decades, there has been a notable increase in the incidence of precocious puberty across countries. For instance, in Denmark, the annual incidence of precocious puberty among girls younger than 8 years rose from 2.6 to 14.6 cases per 10,000 individuals between 1998 and 2017 [2]. Similar increasing trends have also been observed in a few other countries, such as South Korea, where the incidence among girls younger than 9 years rose 4.7 times between 2008 and 2014 [3], and Spain, where the incidence among girls younger than 8 years increased 16.7 times between 1997 and 2006 [4]. Among these countries, China stands out because the incidence is increasing even more rapidly and there is a larger population base [5]. This raises the urgent necessity of clarifying risk factors to facilitate efficient health promotion strategies. Certain factors or preexisting health conditions are speculated to explain the rising incidence of precocious puberty, including genetic predisposition, obesity, environmental exposures, and socioeconomic conditions [1,6-8]. However, specific risk factors involved in the pathogenesis of precocious puberty and their potential interactions still remain unclear and underexplored.

Air pollution is the second leading cause of global disease burden, and exposure to fine particulate matter (PM_2.5_) could underlie most cases of air pollution–related disease, considering the complex toxicity of its components and the fact that over 90% of the world’s population lives in highly polluted locations [9]. Some PM_2.5_ components, such as phthalate, bisphenol analogs, and polycyclic aromatic hydrocarbons (PAHs), have been recognized as endocrine-disrupting chemicals (EDCs), affecting the normal production and signaling of sex hormones [10-12]. Some previous studies explored the association between long-term exposure to PM_2.5_ and the risk of precocious puberty or related symptoms in children, but the conclusions remained inconsistent [7,13-15]. Explanations may be related to differing susceptibility across populations and the varied concentration of the essential components of PM_2.5_ across countries and regions.

Ambient PM_2.5_ pollution is a significant environmental hazard in China. Although air pollution has decreased dramatically in the past decade due to a series of new laws and advancements in environmental protection, the annual mean PM_2.5_ concentration in 2023 was still 5 times higher than the recommended threshold of the World Health Organization (WHO) Air Quality Guidelines [9,16]. Considering the substantial number of children and the increasingly early age at onset of puberty, the adverse impact of PM_2.5_ on precocious puberty, if it indeed exists, could stand out as a significant public health concern in China. However, to date, limited studies have clarified this research question.

Using data from a survey covering 30 cities of 11 provinces, our study aims to estimate the association between long-term exposure to PM_2.5_ components and precocious puberty among school-aged children and to identify the components with the highest effect size in China. This will be indispensable for understanding the harmful effects of PM_2.5_ and for health care promotion and environmental protection in this country.

## Methods

### Study Population

We analyzed data from eligible-aged children in the Prevalence and Risk Factors for Obesity and Diabetes in Youth (PRODY) study. A detailed profile of the PRODY data has been described previously [17]. Briefly, PRODY is a multicenter cross-sectional study conducted among children aged 3 to 18 years in China from 2017 to 2019. Participants were recruited using a stratified cluster random sampling strategy by considering their geographic region, urbanization level, and economic level. Questionnaires and physical examinations were used to collect demographic characteristics, family information, dietary and behavioral habits, growth and development status, and other information, such as weight, height, parental educational level (low: high school or below; high: bachelor or higher), annual family income (first quartile [Q1]: <50,000 yuan [1 yuan=US $0.14]; Q2: 50,000‐100,000 yuan; Q3: 100,000‐200,000 yuan; Q4: >200,000 yuan), physical activity time (Q1:<90 min/week; Q2: 90‐150 min/week; Q3:>150 min/week), frequency of consumption of dairy products, meat, fried foods, soy products, and junk foods (Q1:<1 times/month; Q2: 1‐2 times/month; Q3: 3‐4 times/month; Q4:>4 times/month), whole grains (Q1:<1 times/week; Q2: 1‐2 times/week; Q3: 3‐4 times/week; Q4:>4 times/week), and the consumption of sugary drinks, sweet fruit, and acidic fruit (yes or no). BMI (in kg/m^2^) was calculated according to the growth reference of the WHO [18] and then categorized into 2 groups: low (normal weight or below) and high (overweight or obese).

**Figure 1.**
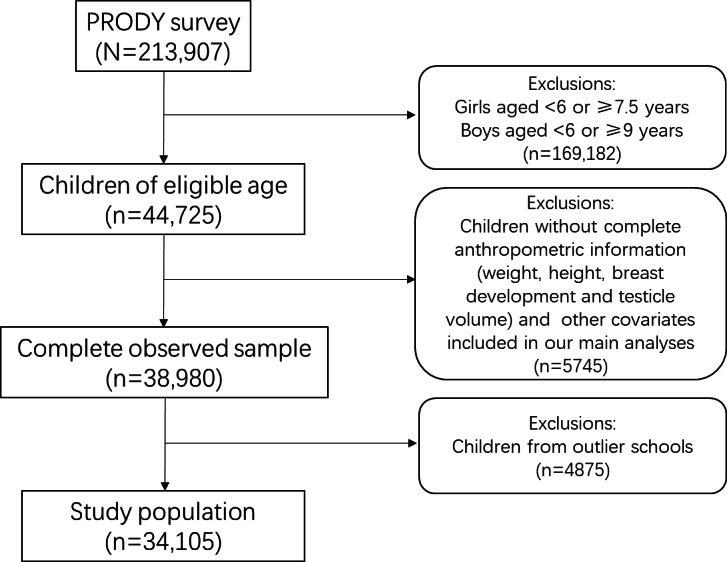
Flowchart of the inclusion and exclusion of study population. PRODY: Prevalence and Risk Factors for Obesity and Diabetes in Youth.

This study initially included 213,907 participants across 11 provinces, autonomous regions, or municipalities in China (Xinjiang Uyghur Autonomous Region, Henan, Hubei, Guangdong, Guangxi Zhuang Autonomous Region, Zhejiang, Shanghai, Fujian, Jilin, Beijing, and Tianjin). Among the participants, 44,725 were at the eligible age for studying precocious puberty, and 38,980 of these (87.2%) accepted the related physical examination. Inclusion and exclusion criteria for the participants are shown in Figure 1. Briefly, based on previous studies and the diagnostic criteria for precocious puberty in China [5,19], we selected girls aged 6 to 7.5 years and boys aged 6 to 9 years as the study population. Children without complete anthropometric information or necessary covariates were excluded. To ensure the robustness of the analysis, schools were excluded as outliers if their prevalence rate of precocious puberty was 1.5 times greater or less than the IQR of the prevalence rates across all schools. The sample size for final analysis was 34,105. Differences in the main demographic characteristics of the study population and the original population were not statistically significant (Table S1 in Multimedia Appendix 1).

### Ethical Considerations

This study protocol was approved by the Institutional Review Board of the Children’s Hospital of Zhejiang University School of Medicine (approval number 2016-JRB-018). Written informed consent to participate in this study was provided by the participants’ legal guardians or next of kin. We intended to use survey weights, but this possibility was denied by the data custodian due to data confidentiality concerns. All data used in this study were nonidentifiable.

### Outcome Measurements

The children’s breast development, testicle volume, and pubic hair were measured by a well-trained endocrinologist. Pubertal stages were assessed following the 5-stage scale described by Marshall and Tanner [20]. Precocious puberty was defined as the initiation of secondary sexual characteristics, designated as Tanner stage 2, before the age of 7.5 years for girls and 9 years for boys [19]. Previous studies have demonstrated that over 98% of Chinese children younger than 6 years have not initiated pubertal development [5]. In line with previous studies, we confined the study population to girls aged 6 to 7.5 years and boys aged 6 to 9 years to improve modeling stability.

### Exposure Measurements

Data on annual average concentrations of PM_2.5_ and its 5 major components, sulfate (SO_4_^2−^), nitrate (NO_3_^−^), ammonium (NH_4_^+^), organic matter (OM), and black carbon (BC), were collected from the Tracking Air Pollution in China database [21] at a spatial resolution of 0.1° × 0.1° for the entire area of China for the period 2012 to 2019. The concentration at each school location was then calculated. Tracking Air Pollution developed a 2-stage machine learning model to predict daily PM_2.5_ concentration using data from PM_2.5_ measurements, satellite aerosol optical depth retrievals, online community multiscale air quality simulations, meteorological reanalysis data, land use information, and population distribution. Methodological details have been described elsewhere, with the data quality also warranted as high [22,23]. The 5-year average concentrations of PM_2.5_ and its 5 major components, calculated as the average over the 0 to 4 years preceding the children’s physical examinations, were assigned to each school address as a surrogate for personal exposure.

### Statistical Methods

#### Single Exposure Model

Mixed-effects logistic regression was applied to quantify the association between PM_2.5_ concentration and each of its 5 components with the binary status of precocious puberty, with the “province” indicator entered as the random intercept. Considering that certain dietary variables were correlated (Figure S1 in Multimedia Appendix 1), we used a least absolute shrinkage and selection operator regression model with the minimum mean squared error to select important dietary confounders related to precocious puberty (Table S2 in Multimedia Appendix 1). The main models were adjusted for gender, age, parental educational level, annual family income, physical activity time, BMI, and dietary habits, including frequency of consumption of dairy products, meat, fried food, and whole grains, as well as the intake of sugary drinks as a snack, fruit as a snack, sweet fruit, and acidic fruit. The results were described as odds ratios (ORs) for precocious puberty with the 95% CI per IQR increase in the concentration of each air pollutant. In this study, PM_2.5_ and its 5 major components were not entered into a single model at the same time to avoid potential multicollinearity problems. The potential interaction effects were examined individually by adding an additional interaction term between PM_2.5_ and each of these variables, including parental educational level, annual family income, physical activity time, BMI, and dietary habits.

#### Joint Exposure Model

Given the high correlations and uncertain relationships among the various components of PM_2.5_, we used a weighted quantile sum (WQS) regression model to assess the joint exposure effects. The WQS model was used to construct a cumulative linear index by grouping PM_2.5_ components into quartiles, with the corresponding weight of each component indicating its contribution to the overall index. WQS regression assumes that all exposures included in the index are associated with the outcome in the same direction. Therefore, we first evaluated the direction of the association between each pollutant and precocious puberty through the single exposure model. As suggested previously, the training and validation datasets were split 40% and 60%, and the bootstrap was set to 1000 times [24].

#### Sensitivity Analyses

A series of sensitivity analyses were conducted to examine the robustness of our main findings. First, the main models were expanded by additionally adjusting for other dietary variables selected from the literature that failed the selection procedure. Second, we modified the personal exposure from 0‐4 years to 1‐4 years and 0‐2 years to explore whether the results might differ with exposure duration. Third, we further adjusted for gaseous pollutants, such as SO_2_, CO_2_, and O_3_. The quantile-based g-computation (QGC) model was used for sensitivity analysis of the joint exposure model, as it allows for the evaluation of combined effects of exposure in different directions [25]. Meta regression was used to test the significance of differences in the results of the main analyses and sensitivity analyses.

R (version 4.3.0; R Foundation for Statistical Computing) was used for analyses. *P* values <.05 were considered statistically significant.

## Results

Table 1 presents the demographic characteristics of the study participants. A total of 34,105 children (n=23,683, 69.44% boys) with an average age of 7.25 (SD 0.82) years were enrolled in this study. Among them, 62.39% (21,279/34,105) had an annual family income exceeding 100,000 yuan (US $14,327), 63.25% (21,570/34,105) had parents with a college degree or higher, and 26.01% (8872/34,105) were categorized as overweight or obese. A total of 401 of the 34,105 children (1.18%) were diagnosed with precocious puberty, of which 214 were boys (0.9%) and 187 were girls (1.79%). The average individual exposure over a 5-year period was 47.93 (SD 16.52) μg/m^3^ for PM_2.5_, 2.31 (SD 0.59) μg/m³ for BC, 6.34 (SD 2.25) μg/m³ for NH_4_^+^, 11.74 (SD 3.75) μg/m³ for OM, 9.52 (SD 3.95) μg/m³ for NO_3_^−^ and 8.74 (SD 2.11) μg/m³ for SO_4_^2−^, respectively (Table S3 in Multimedia Appendix 1). A map of PM_2.5_ concentration and the study population during the study period is shown in Figure S2 in Multimedia Appendix 1.

**Table 1.** Basic characteristics of participants enrolled during 2017 and 2019 in 30 cities in China.

Variables		Precocious puberty (n=401)	Nonprecocious puberty (n=33,704)
PM_2.5_ concentration (μg/m^3^), mean (SD)		47.92 (16.79)	47.93 (16.52)
Age (years), mean (SD)		7.56 (0.85)	7.24 (0.82)
**Gender, n (%)**			
	Female	187 (46.63)	10,235 (30.37)
	Male	214 (53.37)	23,469 (69.63)
**Parental educational level, n (%)**			
	High school or below	180 (44.89)	12,355 (36.66)
	Bachelor or higher	221 (55.11)	21,349 (63.34)
**Annual family income (yuan; 1 yuan=US $0.14), n (%)**			
	<50,000	67 (16.71)	5530 (16.41)
	50,000‐10,0000	98 (24.44)	7131 (21.16)
	100,000‐200,000	120 (29.93)	10,902 (32.35)
	>200,000	116 (28.93)	10,141 (30.09)
**Physical activity (minutes/week) , n (%)**			
	<90	154 (38.4)	13,700 (40.65)
	90‐150	168 (41.9)	12,634 (37.49)
	>150	79 (19.7)	7370 (21.87)
**BMI, n (%)**			
	Underweight or normal	211 (52.62)	25,022 (74.24)
	Overweight or obese	190 (47.38)	8682 (25.76)
**Consumed sugary drinks, n (%)**			
	No	283 (70.57)	25,630 (76.04)
	Yes	118 (29.43)	8074 (23.96)
**Consumed sweet fruit, n (%)**			
	No	71 (17.71)	4709 (13.97)
	Yes	330 (82.29)	28,995 (86.03)
**Consumed acidic fruit, n (%)**			
	No	197 (49.13)	14,884 (44.16)
	Yes	204 (50.87)	18,820 (55.84)
**Junk food frequency (times/month), n (%)**			
	<1	354 (88.28)	30,226 (89.68)
	1‐2	27 (6.73)	2462 (7.30)
	3‐4	15 (3.74)	750 (2.23)
	>4	5 (1.25)	266 (0.79)
**Whole grains frequency (times/week), n (%)**			
	<1	63 (15.71)	5196 (15.42)
	1‐2	248 (61.85)	20,788 (61.68)
	3‐4	71 (17.71)	5636 (16.72)
	>4	19 (4.74)	2084 (6.18)
Dairy product frequency (times/day), mean (SD)		0.66 (0.54)	0.72 (0.57)
Meat frequency (times/day), mean (SD)		2.60 (1.46)	2.73 (1.46)
Fried food frequency (times/day), mean (SD)		0.18 (0.55)	0.14 (0.46)
Soy product frequency (times/day), mean (SD)		0.93 (0.88)	0.98 (0.89)

**Figure 2.**
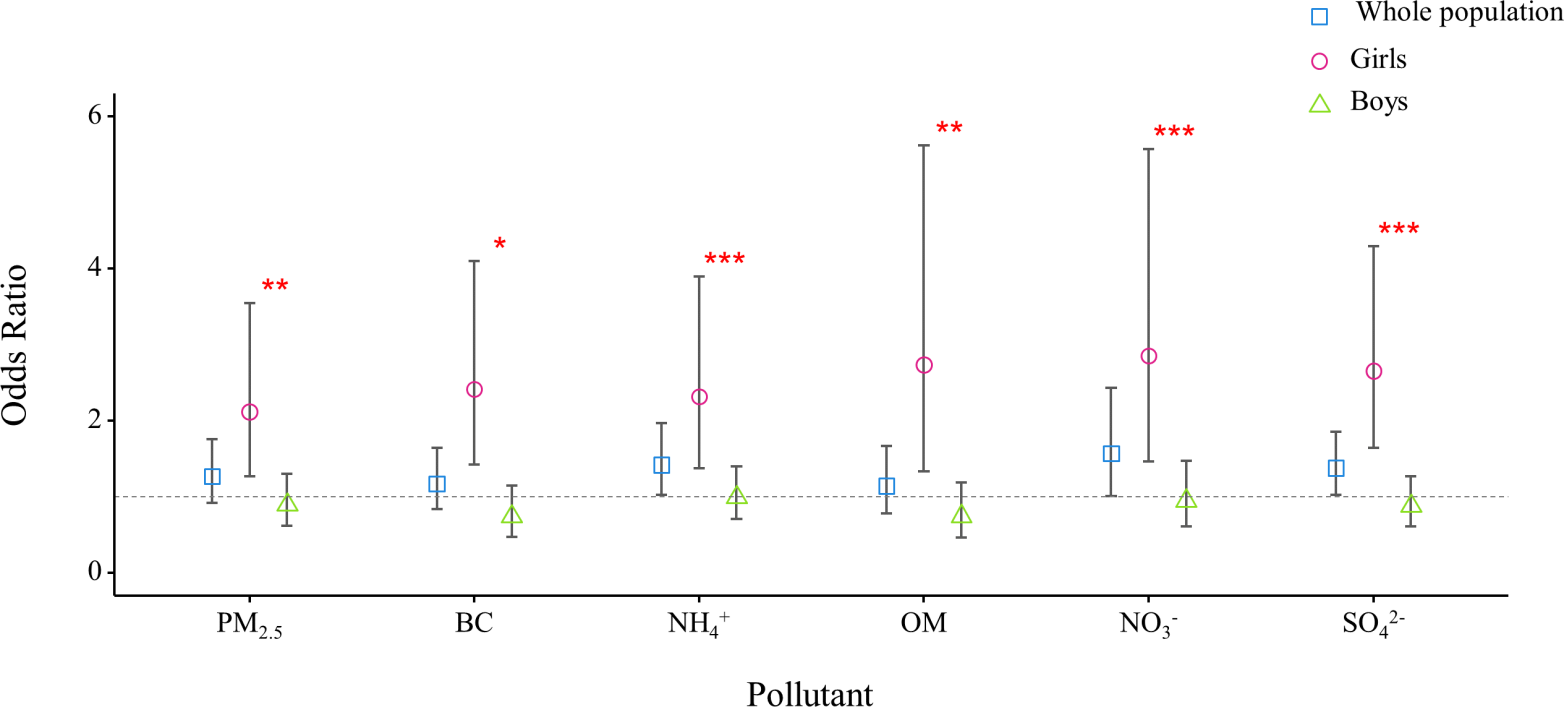
Odds ratio for precocious puberty per IQR increase in PM_2.5_ mass and its 5 major components. PM_2.5_ :fine particulate matter; BC: black carbon; OM: organic matter; NO_3_^−^: nitrate; NH_4_^+^: ammonium; SO_4_^2^−: sulfate. Asterisks indicate the significance of differences between boys and girls: ^*^P<.05, ^**^P<.01, ^***^P<.001.

The analysis of the single exposure model indicated that the ORs for precocious puberty for the whole study population were 1.27 (95% CI 0.92-1.75) for total PM_2.5_ mass, 1.42 (95% CI 1.03‐1.97) for NH_4_^+^, 1.57 (95% CI 1.01‐2.43) for NO_3_^−^, and 1.38 (95% CI 1.03‐1.86) for SO_4_^2_−_^ per IQR increase in their 5-year average concentrations (Figure 2). A clear gender variation in the effect size of PM_2.5_ exposure was observed: per IQR increase in PM_2.5_ mass, the ORs were 2.12 (95% CI 1.27‐3.55) for girls and 0.90 (95% CI 0.62‐1.30) for boys (*P*=.01). In addition, the effect sizes of all 5 PM_2.5_ components were also only significant for girls.

**Figure 3.**
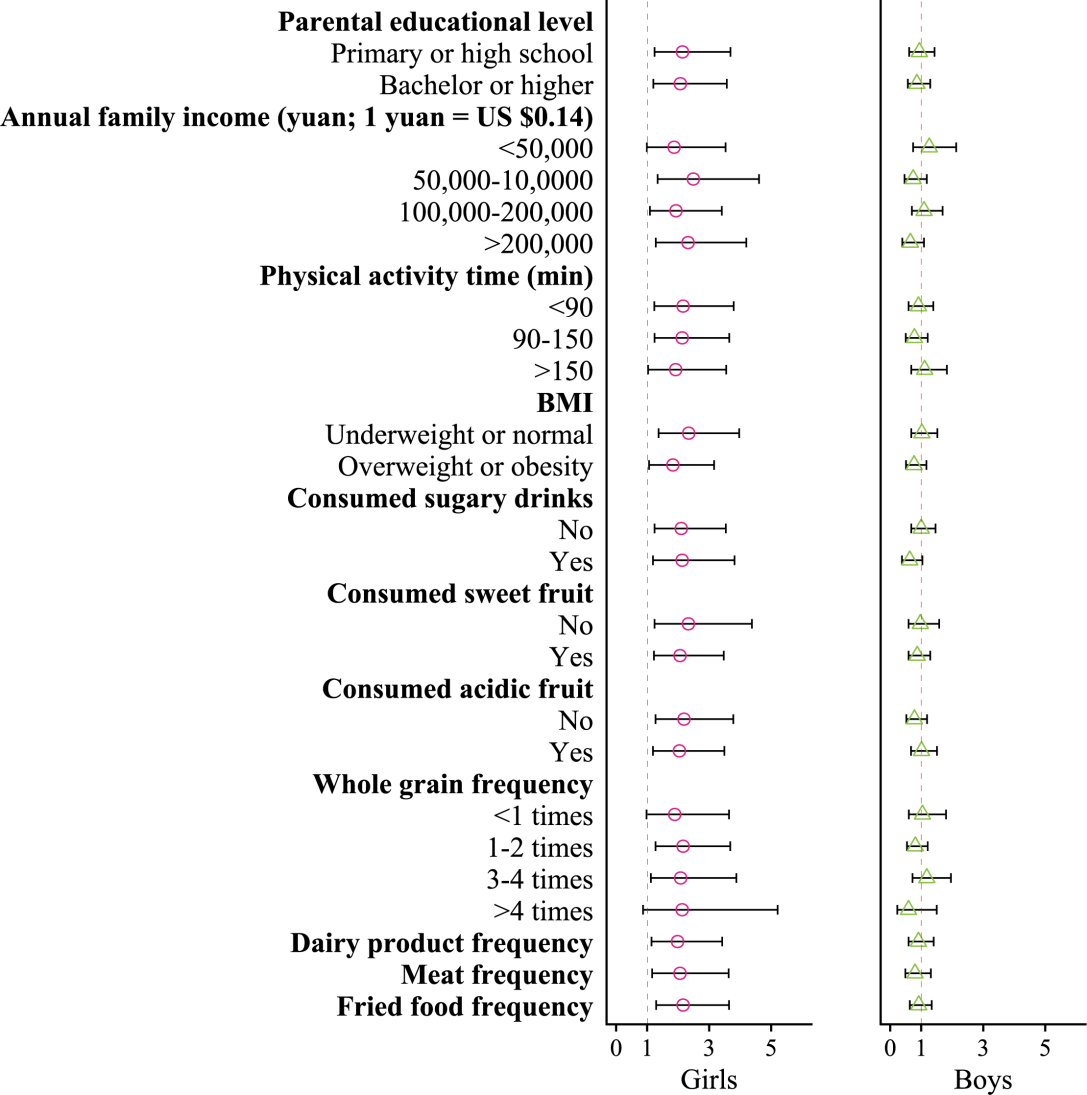
The modification effect of individual characteristics and dietary habits on the association between PM_2.5_ exposure and precocious puberty.

The effect size of PM_2.5_ was nonsignificantly higher in groups with certain demographic characteristics, family information, and behavioral habits (Figure 3). For example, the OR for precocious puberty was 2.49 (95% CI 1.34‐4.61) for girls with a family income at the Q2 level and 1.87 (95% CI 0.99‐3.53) for those with family income at the Q1 level. Similarly, the ORs were 2.34 (95% CI 1.37‐3.97) and 1.83 (95% CI 1.06‐3.16) for groups with low BMI and high BMI, respectively. Certain modifying effects were observed in boys, such as family income (<50,000 yuan group vs >200,000 yuan group; *P*=.03) and sugary drink consumption (no vs yes; *P*=.03).

**Figure 4.**
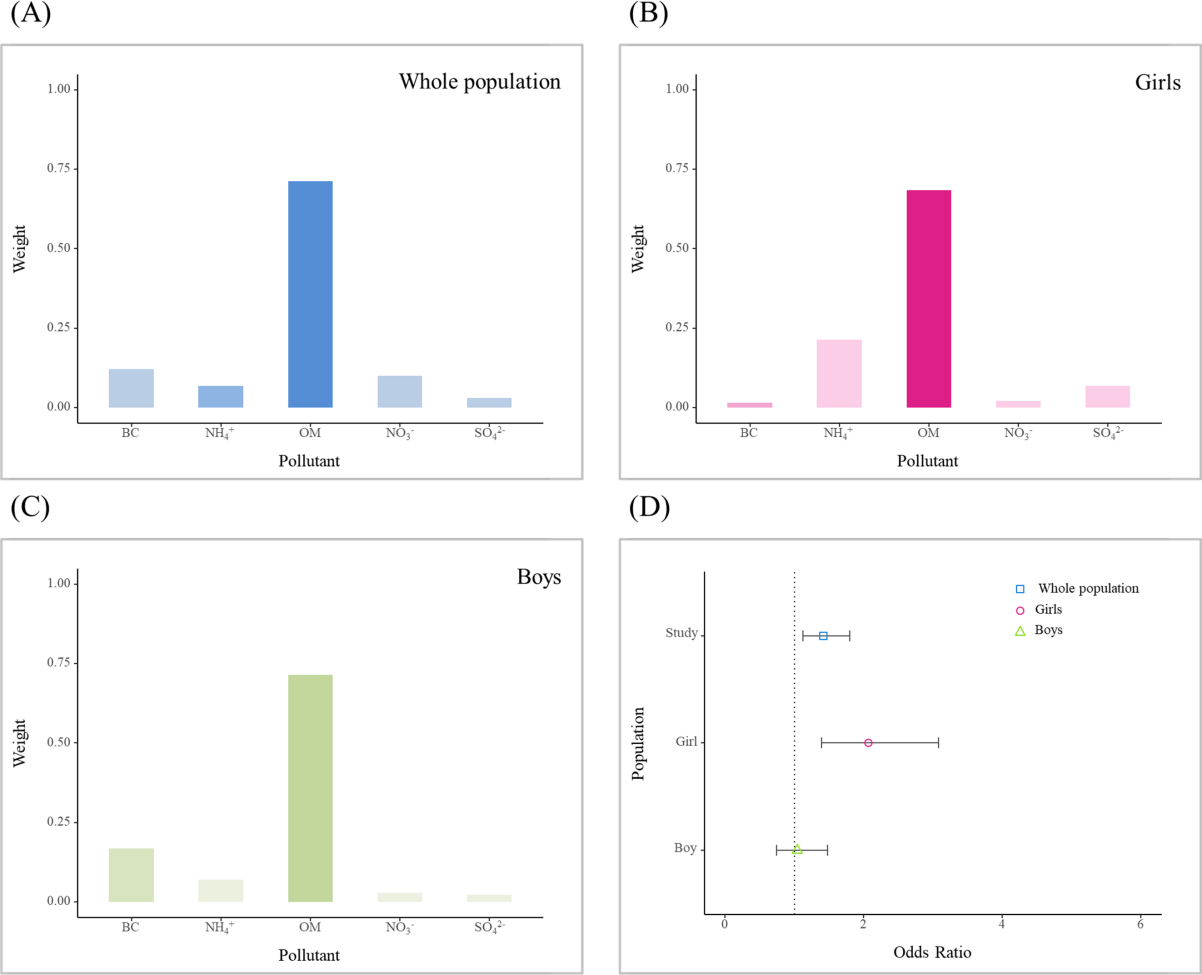
Weights of the contributions of the 5 major components of fine particulate matter (PM_2.5_) to its total effect size. Mean weights for (**A**) the whole study population; (**B**) girls; (**C**) boys. (**D**) Odds ratios for precocious puberty per IQR increase in the weighted quantile sum regression (WQS) index. WQS models were adjusted for gender; age; maximum level of parental education; annual family income; weekly physical activity time; BMI; consumption frequency of dairy products, meat, fried foods, and coarse food grain; intake of sugary drinks, sweet fruit, and acidic fruit; and province. BC: black carbon; OM: organic matter; NO_3_^−^: nitrate; NH_4_^+^: ammonium; SO_4_^2^−: sulfate.

As shown in Figure 4, the WQS index was significantly associated with precocious puberty (OR 1.42, 95% CI 1.12‐1.80). The major contributors were OM, BC, and NO_3_^−^, with their weights in the overall effect size being 0.71, 0.12, and 0.10, respectively. After stratification by gender, a significant association was observed only in girls.

The strength of the association was stable after adjusting for additional covariables and gaseous pollutants or changing the exposure period in both the single-air-pollutant models and WQS models. The association between PM_2.5_ components and precocious puberty remained unchanged in the QGC model, with OM continuing to be the major positive contributor to the overall effect of total PM_2.5_ mass (Tables S4-5 in Multimedia Appendix 1).

## Discussion

### Principal Results

This study explored the association between exposure to PM_2.5_ and its major components with precocious puberty based on a large survey of school-aged children in China. We observed that PM_2.5_ mass was significantly associated with precocious puberty only in girls. OM was detected as the major effective component when the 5 major components of PM_2.5_ were jointly exposed. Modification effects of certain factors at the individual or family levels were observed (eg, family income and sugary drink consumption), especially for boys.

### Comparison With Prior Work

Recent evidence suggests that there is significant variation in the prevalence of precocious puberty across regions and study periods; the prevalence ranges from <0.05% to 2.53% in Denmark, Korea, and China [15,26,27]. To our knowledge, our PRODY study is to date the only large-scale survey on precocious puberty in China. We found that the prevalence in the study areas was 0.9% in boys and 1.79% in girls. Previously, only one study (the predecessor of PRODY) investigated precocious puberty in 5 Chinese provinces and reported a prevalence of 0.43% [26]. The differing prevalence across studies may be partly explained by differences in diagnostic criteria, population characteristics, the years of investigation, and certain limitations in sampling.

Previously, some studies have explored the effect of long-term PM_2.5_ exposure on pubertal development or related sexual hormones and obtained inconsistent results. A cohort study in Poland revealed that children exposed to an average annual concentration of PM_2.5_ exceeding 25 μg/m^3^ were at high risk of menarche occurring before the age of 11 years [14]. Similarly, Jung et al [28] found that each 1 μg/m^3^ increase in the 3-year average of annual mean PM_10_ concentration was associated with age acceleration at menarche by 0.031 years in 639 girls aged 13 to 17 years in South Korea. Additionally, a recent large-scale (n=1,205,784) nationwide retrospective cohort study reported that 4-year exposures to PM_2.5_ and PM_10_ were associated with precocious puberty in girls in South Korea [15]. By contrast, in Hong Kong’s cohort of children born in 1997, Huang et al [7] found a nonsignificant association between childhood PM_10_ exposure and precocious puberty. A German study reported a weak association between PM_2.5_ exposure and decreased estradiol level in girls in Munich [13].

There may be several explanations for the inconsistent findings across studies. For example, in comparison to the Hong Kong study, which used the age of 11 years as the diagnostic threshold for pubertal stage, our study applied a younger diagnostic age (<7.5 years for girls and <9 years for boys), following the latest official recommendation in mainland China. This lower threshold may more effectively capture the early onset of pubertal development in children and the related effect size of PM_2.5_. In addition, the inconsistent results of these studies may also be attributed to the differences in study area, population characteristics, outcome definitions, and other potential confounders like socioeconomic status, as well as exposure period and levels. This study provides supporting evidence for the adverse effect of childhood PM_2.5_ exposure on precocious puberty among a nationwide sample in China.

The potential mechanisms underpinning the adverse effect of PM_2.5_ on precocious puberty have been rarely discussed. One suggested hypothesis is the endocrine disruption caused by PM_2.5_. Certain EDCs, such as phthalates, bisphenol analogs, and PAHs, have been detected as PM_2.5_ components in several major Chinese cities [29-31]. These chemicals may target the estrogen receptor or kisspeptin in the hypothalamic nuclei and exhibit estrogenic or antiandrogenic activity, thereby promoting hypothalamic maturation and consequently causing precocious puberty [8,32-34]. In addition, some heavy metals found in PM_2.5_, especially lead and cadmium, have also been proven to disrupt estrogen levels in female individuals and influence pubertal development [35,36].

The health effects of PM_2.5_ depend on its chemical components. An increasing number of epidemiological studies have paid attention to the association between PM_2.5_ chemical components and a range of health outcomes, such as pregnancy disorders, lung function, mortality, and body weight [37-40]. To the best of our knowledge, our study is the first to report associations of PM_2.5_ components with precocious puberty. We found that OM is the most important component of PM_2.5_ associated with precocious puberty in China. OM is produced by the incomplete combustion of solid fuels, which have higher toxicity than noncombustion aerosols [41]. In China, OM is the primary component of PM_2.5_ in several major cities [42], posing a significant health risk to the population. Although the differential impact of components of PM_2.5_ on precocious puberty has not yet been adequately examined, some underlying mechanisms may explain the observed effects. As previously mentioned, the chemical components in PM_2.5_ that exert endocrine-disrupting effects are predominantly OM, such as PAHs. In addition, OM has the potential to induce mutagenic, inflammatory, and lipid metabolic disturbances in vivo [43-45]. However, without data at the cellular and molecular levels, it is beyond the scope of this study to speculate further about the potential pathways mediating the impact of OM on pubertal development.

Previous studies have suggested that the effects of PM_2.5_ on children’s pubertal development may differ by gender [13,15]. Consistent with this, our study observed a more significant association between PM_2.5_ and precocious puberty in girls than in boys. It has been speculated that gender differences are due to the varying effects of PM exposure on different hormone levels in vivo [46], as well as the fact that prepubertal girls are particularly sensitive to low levels of exogenous estrogens [47]. In addition, gender differences may be related to the criteria for measuring secondary sexual characteristics, which depend on physical examinations of breast development and pubic hair in girls and testicle volume and pubic hair in boys, rather than on sex hormones and gonad B-ultrasounds.

Significant modifying effects of family income and sugary drink consumption were observed. Previously, several studies explored potential modifiers of the adverse effect of long-term PM_2.5_ exposure, although few have focused on pubertal development. For example, a large cohort study in Canada found that the impact of PM_2.5_ on mortality was greater for low-income earners than for high-income earners [48]. A study in the United States indicated that the cardiovascular risks associated with PM_2.5_ exposure were higher among low-income groups compared to high-income groups [49]. The inequalities in PM_2.5_ exposure between high-income populations and low-income populations may partly explain the modifying effect of family income.

Pubertal timing is sensitive to nutritional regulation; for example, girls with the highest levels of dietary isoflavone intake may experience early onset of breast development [50]. Some animal studies have found that fructose diet–fed rats had lower plasma testosterone levels, which can lead to a high sensitivity of the pituitary-gonadal axis in Leydig cells [51]. It can be speculated that dietary factors may potentially involve pathways that overlap with PM_2.5_ and promote or hinder the effects of PM_2.5_ on precocious puberty. However, there is still a lack of evidence to explain why significant modifying effects of family income and sugary drink consumption were observed more prominently in boys than in girls. Due to the absence of detailed information, further exploration is not feasible. Future studies may be able to elucidate the underlying mechanisms.

### Strengths and Limitations

This study has several strengths that can be mentioned. First, our findings came from a survey covering 30 cities in 11 provinces, improving the generalizability when compared to studies with a smaller geographical scale, such as a single city. Second, the PRODY survey incorporated measurements of breast development and testicle volume conducted by medical professionals, resulting in a more accurate diagnosis of precocious puberty compared to studies using self-reported or parent-reported data. Third, we analyzed the effects of joint exposure to PM_2.5_ components on precocious puberty using WQS regression, which can identify major contributors and therefore provide targeted recommendations for air quality–related health improvement.

We acknowledge some limitations of our analysis. In this study, air pollutant concentrations at the school level may not fully reflect all sources of air pollution, such as indoor air pollution within households, potentially leading to exposure misclassification. Because of the large sample size in this study and the limitations imposed by survey costs, we were unable to measure children’s air pollution exposure at a finer scale. Future studies are strongly encouraged to consider a more holistic approach by incorporating direct measurements of indoor air quality, possibly using portable air monitoring devices. Due to ethical considerations, only children willing to undergo physical examinations were included in the study, which might have introduced a certain sampling bias. Without access to data on appropriate survey weights, we were unable to evaluate the accurate generalization of our findings to the whole target population of the country, which warrants further exploration when data are available in the future. Due to the cross-sectional study design, the inference of causality was limited. Furthermore, owing to constraints in data availability, our study did not account for the adverse effects of some other toxic components (ie, heavy metals) or confounding variables such as parental history of precocious puberty, sleep patterns, and psychological status. We suggest that future research should include these additional variables to better understand their potential impacts on the results.

### Conclusion

Our study suggests that OM may be a major contributor to the association between long-term exposure to PM_2.5_ and precocious puberty. Our findings further endorse the call for reducing fossil fuel emissions, concurrently advocating the development of specific and effective strategies to protect children’s developmental health.

## Supplementary material

10.2196/62861 Multimedia Appendix 1 Supplementary materials showing the distribution of air pollutant concentrations and the study population during the study period, a comparison of characteristics between the original recruited population and the study population, and the results of the sensitivity analysis.

## Abbreviations

BC: black carbon
EDC: endocrine-disruptor chemical
NH_4_^+^: ammonium
NO_3_^-^: nitrate
OM: organic matter
OR: odds ratio
PAH: polycyclic aromatic hydrocarbon
PM_2.5_: fine particulate matter
PRODY: Prevalence and Risk Factors for Obesity and Diabetes in Youth
Q: quartile
QGC: quantile-based g-computation
SO_4_^2-^: sulfate
WHO: World Health Organization
WQS: weighted quantile sum
