# Supplementary material for: Long-Term Exposure to Fine Particulate Matter (PM2.5) Components and Precocious Puberty Among School-Aged Children: Cross-Sectional Study
Source: JMIR Public Health Surveill. 2025 Feb 7;11:e62861. doi: 10.2196/62861 (PMC11830487; doi:10.2196/62861)
Supplement: Multimedia Appendix 1 [file publichealth-v11-e62861-s001.docx]

# Supplementary materials

Long-term exposure to PM_2.5_ components and precocious puberty among school-aged children in China: A Cross-sectional Study

**Table of contents**

**1.Table S1:** Comparison of characteristics between the original and the study population

**2.Table S2:** Estimates of the lasso regression model

**3.Table S3:** Air pollutant concentrations summarized by city

**4.Table S4:** Sensitivity analyses of single air pollutant models

**5.Table S5:** Sensitivity analyses of WQS models

**6. Figure S1:** Correlation heatmap of dietary factors

**7. Figure S2:** Map of PM_2.5_ concentration and locations of study population during the study period

Table S1: Comparison of characteristics between the original and the study population

| Variables | | Study population  (N = 34,105) | Original population (N = 38,980) | *P* |
| --- | --- | --- | --- | --- |
| **Gender** | |  |  | 0.55 |
|  | Female | 10422 (30.56%) | 11831 (30.35%) |  |
|  | Male | 23683 (69.44%) | 27149 (69.65%) |  |
| **Parental educational level** | |  |  | <0.01 |
|  | high school or below | 12535 (36.75%) | 14915 (38.26%) |  |
|  | Bachelor or higher | 21570 (63.25%) | 24065 (61.74%) |  |
| **Annual family income** | |  |  | 0.03 |
|  | <50,000 yuan | 5597 (16.41%) | 6895 (17.69%) |  |
|  | 50,000-10,0000 yuan | 7229 (21.20%) | 8440 (21.65%) |  |
|  | 100,000-200,000 yuan | 11022 (32.32%) | 12232 (31.38%) |  |
|  | >200,000 yuan | 10257 (30.07%) | 11413 (29.28%) |  |
| **Physical activity time** | |  |  | 0.06 |
|  | <90 minutes/week | 13854 (40.62%) | 15625 (40.08%) |  |
|  | 90-150/week | 12802 (37.54%) | 14906 (38.24%) |  |
|  | >150 minutes | 7449 (21.84%) | 8449 (21.68%) |  |
| **BMI** | |  |  | 0.09 |
|  | Underweight or normal | 25233 (73.99%) | 28619 (73.42%) |  |
|  | Overweight or obesity | 8872 (26.01%) | 10361 (26.58%) |  |
| **Sugary drinks in snacks** | |  |  | 0.20 |
|  | Not | 25913 (75.98%) | 29458 (75.57%) |  |
|  | Had | 8192 (24.02%) | 9522 (24.43%) |  |
| **Sweet fruits** | |  |  | 0.22 |
|  | Not | 4780 (14.02%) | 5587 (14.33%) |  |
|  | Had | 29325 (85.98%) | 33393 (85.67%) |  |
| **Acidic fruits** | |  |  | 0.97 |
|  | Not | 15081 (44.22%) | 17230 (44.20%) |  |
|  | Had | 19024 (55.78%) | 21750 (55.80%) |  |
| **Junk food frequency** | |  |  | 0.84 |
|  | <1 times/month | 15150 (44.42%) | 17489 (44.97%) |  |
|  | 1-2 times/month | 16397 (48.08%) | 18520 (47.62%) |  |
|  | 3-4 times/month | 2062 (6.05%) | 2326 (5.98%) |  |
|  | > 4 times/month | 496 (1.45%) | 558 (1.43%) |  |
| **Coarse food grain frequency** | |  |  | 0.56 |
|  | <1 times/week | 5259 (15.42%) | 6079 (15.60%) |  |
|  | 1-2 times/week | 21036 (61.68%) | 24010 (61.60%) |  |
|  | 3-4 times/week | 5707 (16.73%) | 6491 (16.65%) |  |
|  | > 4 times/week | 2103 (6.17%) | 2400 (6.16%) |  |
| **Dairy products frequency (times/day)** | | 0.72 (0.57) | 0.71 (0.57) | 0.05 |
| **Meat frequency (times/day)** | | 2.72 (1.46) | 2.71 (1.47) | 0.24 |
| **Fried foods frequency (times/day)** | | 0.14 (0.46) | 0.15 (0.47) | 0.15 |
| **Soy products frequency (times/day)** | | 0.98 (0.89) | 0.96 (0.89) | 0.01 |
| **PM_2.5_ (μg/m³)** | | 47.93 (16.52) | 48.07 (16.41) | 0.24 |
| **SO_4_^2-^ (μg/m³)** | | 8.74 (2.11) | 8.76 (2.09) | 0.28 |
| **NO_3_^-^ (μg/m³)** | | 9.52 (3.95) | 9.51 (3.94) | 0.76 |
| **NH_4_^+^ (μg/m³)** | | 6.34 (2.25) | 6.36 (2.25) | 0.24 |
| **OM (μg/m³)** | | 11.74 (3.75) | 11.78 (3.73) | 0.10 |
| **BC (μg/m³)** | | 2.31 (0.59) | 2.32 (0.59) | 0.05 |

Note: BMI: body mass index. Data are presented as mean (SD) for continuous variables and number (%) for categorical variables. *P*≥0.05 denotes that the difference in the study population is not statistically significant compared to the original population.

Table S2: Estimates of the lasso regression model

| Variable name | Variable description |  | Coefficient (_*_10^-4^) |
| --- | --- | --- | --- |
| Vegetable | Vegetable frequency (daily) |  | . |
| Dairy_products | Dairy products frequency (daily) |  | -4.67 |
| Eggs | Eggs frequency (daily) |  | . |
| Seafood | Seafood frequency (daily) |  | . |
| Aquatic_products | Aquatic products frequency (daily) |  | . |
| Organ_meat | Animal organs frequency (daily) |  |  |
| Meat | Meat frequency (white meat and red meat, daily) |  | -0.355 |
| Sugary_drinks | Sugary drinks frequency (daily) |  | . |
| Mushroom | Mushroom and algae frequency (daily) |  | . |
| Fired_foods | Fried foods frequency (daily) |  | 0.96 |
| Soy_products | Soy products frequency (daily) |  | . |
| Staple_food | Staple food frequency (daily) |  | . |
| Sugary_drinks_s | Sugary drinks in snacks | had | 7.3 |
| sweets_s | Confectionery in snacks | had |  |
| Biscuits_s | Biscuits in snacks | had | . |
| Barbecue_s | Barbecue, fried, puffed food in snacks | had | . |
| Pickled_products_s | Pickled products in snacks | had | . |
| Nuts_s | Nuts in snacks | had | . |
| Fruits_s | Fruits in snacks | had | -0.55 |
| Milk_s | Milk and dairy products in snacks | had | . |
| Water_d | Plain water or bottled water in drinks | had | . |
| Juice_d | Pure fruit or vegetable juice | had | . |
| Milk_d | Milk in drinks | had | . |
| Coffee_d | Coffee or tea in drinks | had | . |
| Carbonated_d | Carbonated beverages in drinks | had | . |
| Sweet_fruits | Consume Sweet fruits | had | -4.55 |
| Acidic_fruits | Consume Acidic fruits | had | -2.98 |
| Mild_fruits | Consume mild fruits | had | . |
| Junk_food | Junk food frequency (monthly) | <1 times | . |
|  |  | 3-4 times | . |
|  |  | >4times | . |
| Coarse_food | Coarse food grain frequency (weekly) | <1 times | . |
|  |  | 3-4 times | . |
|  |  | >4times | 4.18 |

Note: ‘.’ denote that the coefficient is shrunk to 0.

Table S3: Air pollutant concentrations summarized by city

| City | PM_2.5_ | SO_4_^2-^ | NO_3_^-^ | NH_4_^+^ | OM | BC |
| --- | --- | --- | --- | --- | --- | --- |
| Wuhan | 57.92 | 10.26 | 12.23 | 7.29 | 14.76 | 2.86 |
| Lishui | 31.02 | 6.51 | 5.91 | 4.73 | 8.25 | 1.90 |
| Nanning | 37.33 | 7.40 | 6.63 | 5.15 | 9.83 | 1.98 |
| Shenzhen | 36.08 | 7.41 | 4.30 | 3.38 | 9.95 | 2.16 |
| Beijing | 76.19 | 10.86 | 14.65 | 9.07 | 18.38 | 3.18 |
| Fuzhou | 27.93 | 5.99 | 4.56 | 3.25 | 8.03 | 1.74 |
| Hangzhou | 47.10 | 9.42 | 10.31 | 6.94 | 10.31 | 2.12 |
| Tianjin | 72.73 | 11.69 | 14.25 | 9.28 | 17.90 | 3.36 |
| Luohe | 64.10 | 12.03 | 16.66 | 10.55 | 14.13 | 2.59 |
| Anyang | 70.32 | 11.71 | 16.23 | 10.31 | 16.47 | 3.01 |
| Jinhua | 40.62 | 8.18 | 8.54 | 6.22 | 9.39 | 1.92 |
| Taizhou | 28.46 | 6.02 | 5.56 | 4.03 | 7.32 | 1.62 |
| Urumqi | 65.10 | 9.05 | 8.74 | 6.02 | 11.61 | 2.69 |
| Shanghai | 42.57 | 8.31 | 8.23 | 5.46 | 9.23 | 2.04 |
| Quzhou | 36.69 | 7.59 | 7.99 | 5.74 | 8.97 | 1.68 |
| Xinxiang | 86.50 | 15.07 | 20.47 | 13.19 | 18.76 | 3.53 |
| Liuzhou | 51.34 | 10.29 | 9.44 | 7.45 | 13.44 | 2.78 |
| Hotan | 54.30 | 3.24 | 4.01 | 2.54 | 8.30 | 1.30 |
| Shaoxing | 42.54 | 8.96 | 9.57 | 6.66 | 9.51 | 2.01 |
| Shangqiu | 68.14 | 12.91 | 17.98 | 11.41 | 15.04 | 2.85 |
| Pingdingshan | 61.65 | 11.36 | 15.09 | 9.92 | 13.65 | 2.48 |
| Huzhou | 43.55 | 8.61 | 10.55 | 6.94 | 9.29 | 1.73 |
| Luoyang | 69.16 | 11.88 | 16.02 | 10.15 | 15.52 | 2.89 |
| Zhengzhou | 81.45 | 13.94 | 18.69 | 11.66 | 18.26 | 3.38 |
| Xiamen | 26.84 | 5.44 | 3.98 | 2.74 | 8.47 | 1.72 |
| Siping | 43.85 | 7.37 | 9.79 | 6.32 | 10.63 | 1.97 |
| Ningbo | 32.72 | 6.72 | 7.37 | 4.95 | 7.57 | 1.53 |
| Changchun | 51.23 | 7.45 | 8.80 | 5.66 | 13.02 | 2.49 |
| Dongguan | 35.45 | 7.44 | 4.80 | 3.71 | 9.73 | 2.17 |
| Puyang | 74.58 | 13.12 | 18.31 | 11.60 | 17.20 | 3.18 |
| Overall | 47.93 | 8.74 | 9.52 | 6.34 | 11.74 | 2.31 |

Units: μg/m^3^

Table S4: Sensitivity analyses of single air pollutant models

| Model settings | Population | Exposure period | OR（95%CI） | *P* |
| --- | --- | --- | --- | --- |
| Main model | Study population  (N=34,105) | 0-4yr | 1.27 (0.92, 1.75) | Ref. |
| Crude model | Study population  (N=34,105) | 0-4yr | 1.28 (0.93, 1.76) | 0.99 |
| Extended model | Study population  (N=34,105) | 0-4yr | 1.27 (0.92, 1.75) | 1.00 |
| Gaseous model | Study population  (N=34,105) | 0-4yr | 1.42 (0.96, 2.11) | 0.67 |
| Main model | Study population  (N=34,105) | 0-2yr | 1.32 (0.99, 1.77) | 0.62 |
| Main model | Study population  (N=34,105) | 1-4yr | 1.26 (0.92, 1.73) | 0.93 |

Note: Crude models were adjusted for gender, age, maximum level of parental education, annual family income, weekly physical activity time, BMI; Main models were adjusted for gender, age, maximum level of parental education, annual family income, weekly physical activity time, BMI, frequencies of dairy products, meats, fired foods and coarse food grain, and the intake of sugary drinks, sweet fruits, acidic fruits; Extended models were adjusted for frequency of junk foods, soy products in addition to main models; Gaseous models were adjusted for SO_2_, CO_2_, and O_3_ in addition to main models. P≥0.05 denotes that the difference in meta-regression model is not statistically significant compared to the control group.

Table S5: Sensitivity analyses of WQS models

| Model settings | Population | Exposure period | OR（95%CI） | Mean weights | | | | | *P* |
| --- | --- | --- | --- | --- | --- | --- | --- | --- | --- |
|  |  |  |  | BC | NH_4_^+^ | NO_3_^-^ | OM | SO_4_^2-^ |  |
| Main model | Study population (N=34,105) | 0-4yr | 1.42(1.12, 1.80) | 0.12 | 0.07 | 0.10 | 0.71 | 0.00 | Ref. |
| Crude model | Study population (N=34,105) | 0-4yr | 1.42(1.12, 1.81) | 0.13 | 0.07 | 0.10 | 0.70 | 0.00 | 0.99 |
| Extended model | Study population (N=34,105) | 0-4yr | 1.42(1.12, 1.81) | 0.12 | 0.06 | 0.12 | 0.70 | 0.00 | 1.00 |
| Gaseous model | Study population (N=34,105) | 0-4yr | 1.47(1.13, 1.91) | 0.28 | 0.07 | 0.06 | 0.60 | 0.00 | 0.84 |
| Main model | Study population (N=34,105) | 0-2yr | 1.39(1.08, 1.78) | 0.21 | 0.19 | 0.00 | 0.60 | 0.00 | 0.88 |
| Main model | Study population (N=34,105) | 1-4yr | 1.41(1.11, 1.79) | 0.07 | 0.07 | 0.12 | 0.73 | 0.00 | 0.96 |
| QGC model | Study population (N=34,105) | 0-4yr | 1.45(1.15, 1.84) | -0.38 | -0.32 | +0.43 | +0.57 | -0.29 | 0.91 |

Note: Crude models were adjusted for gender, age, maximum level of parental education, annual family income, weekly physical activity time, BMI and provinces; Main models were adjusted for gender, age, maximum level of parental education, annual family income, weekly physical activity time, BMI, frequencies of dairy products, meats, fired foods and coarse food grain, the intake of sugary drinks, sweet fruits, acidic fruits and provinces; Extended models were adjusted for frequency of junk foods, soy products in addition to main models; Gaseous models were adjusted for SO_2_, CO_2_, and O_3_ in addition to main models. In QGC model, “+” denotes positive weights, “-” denotes negative weights. P≥0.05 denotes that the difference in meta-regression model is not statistically significant compared to the control group.

Figure S1: Correlation heatmap of dietary confounders


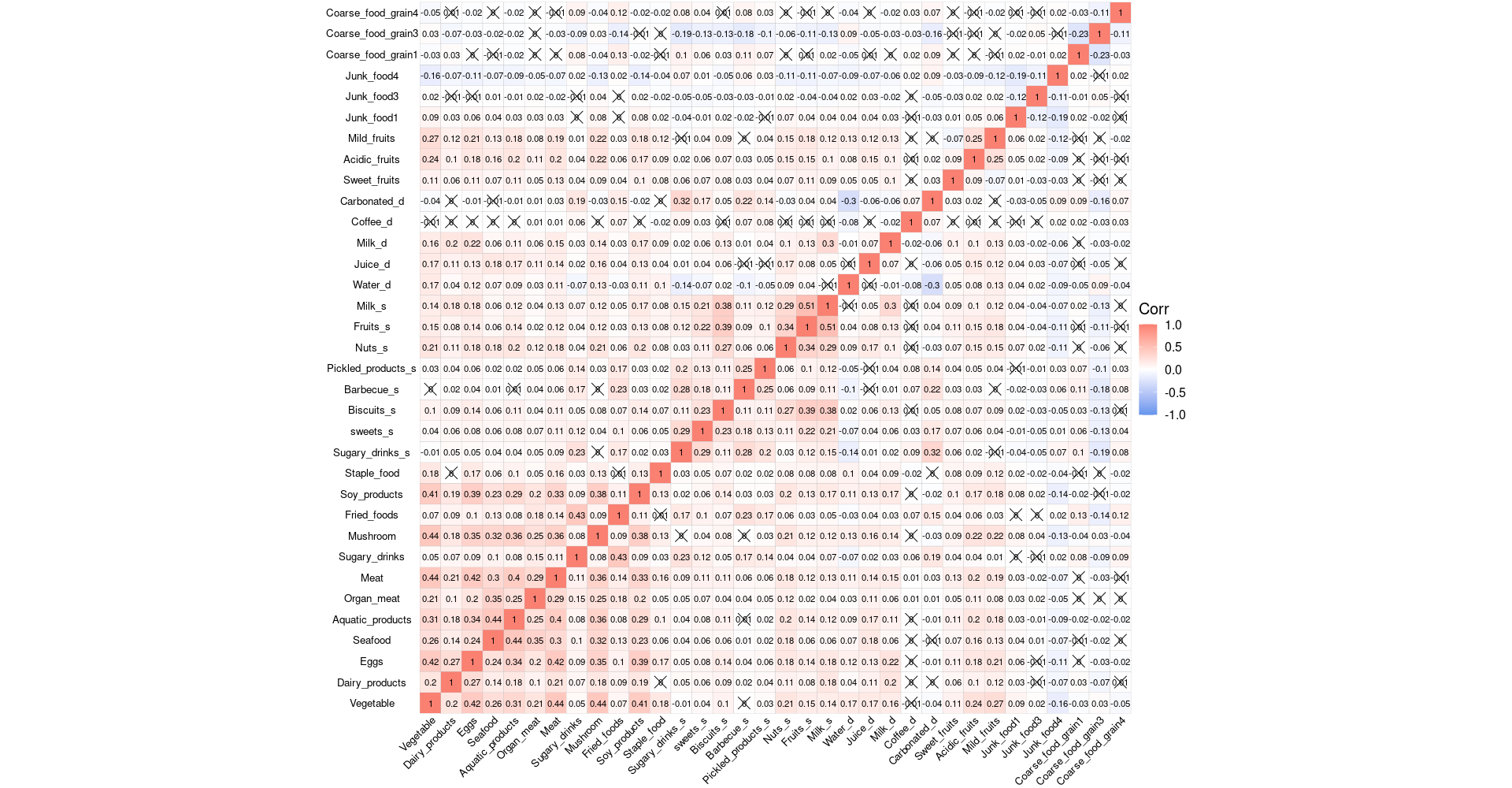


Note: crossed-out boxes denote the *P* of correlation ≥0.05.

Figure S2: Map of PM_2.5_ concentration and study population during the study period


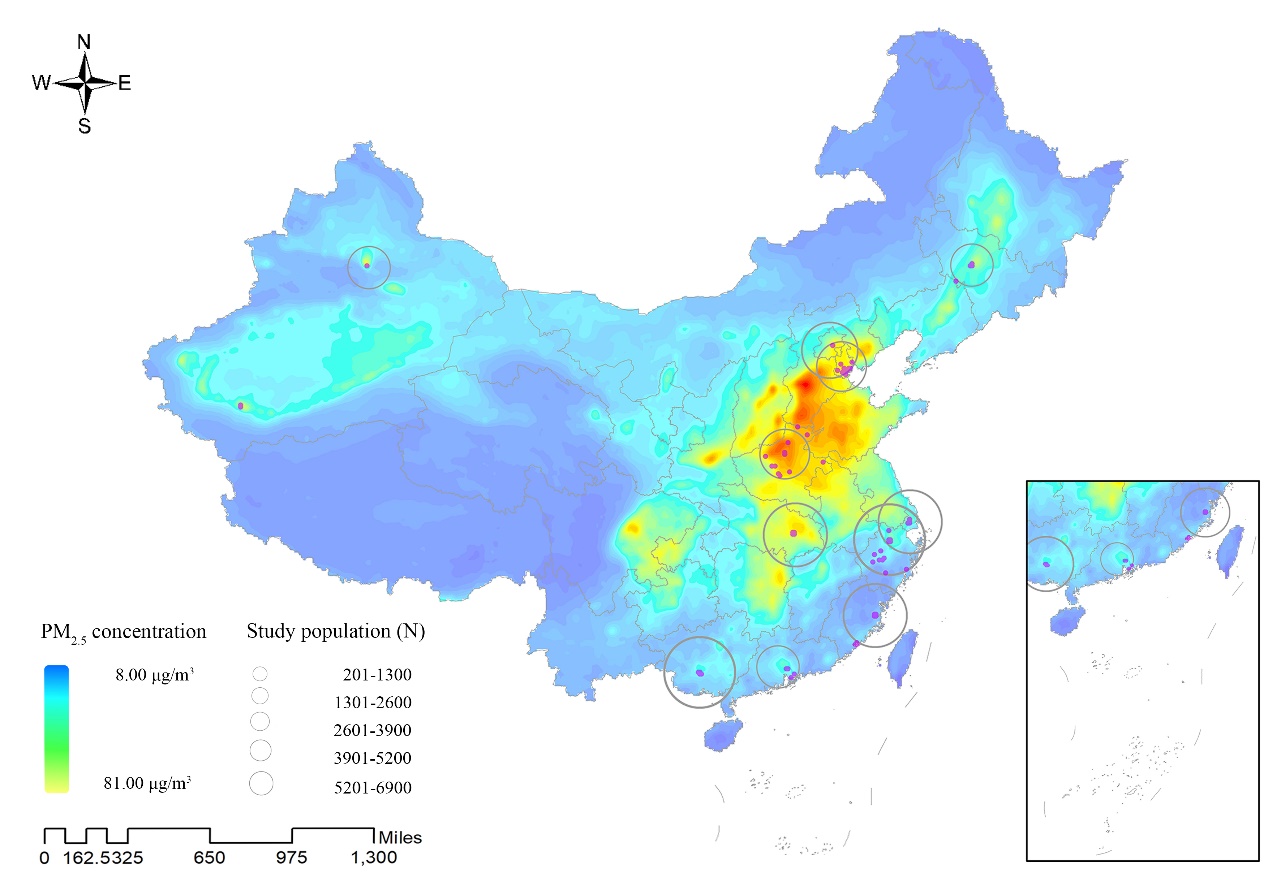


Note: Small islands or areas with missing data are not displayed in this map. The size of circle represents the sample size of the province and the purple dots indicate the locations of the study schools.
